# *Bacillus cereus* bacteraemia complicated by a brain abscess in a pre-term neonate

**DOI:** 10.1099/acmi.0.000080

**Published:** 2019-11-27

**Authors:** Harsha Samarasekara, Catherine Janto, Vishnu Dasireddy, Adam Polkinghorne, James Branley

**Affiliations:** ^1^​ Department of Microbiology and Infectious Diseases, New South Wales Health Pathology, Nepean Blue Mountains Pathology Service, PO Box 63, Penrith, New South Wales, 2751, Australia; ^2^​ Neonatal Intensive Care Unit, Nepean Hospital, Penrith, New South Wales, Australia; ^3^​ The University of Sydney Nepean Clinical School, Faculty of Medicine and Health, University of Sydney, Penrith, New South Wales, 2751, Australia

**Keywords:** Infection, Sepsis, Meningitis, Imaging, Clinical microbiology

## Abstract

*Bacillus cereus* is a common laboratory and environmental contaminant. Reports of severe infections are mainly limited to immunocompromised individuals. In reported cases, the time interval between bacteraemia and neuro-invasion appears to be very short, highlighting the importance of rapid and definitive identification and susceptibility testing of invasive *B. cereus*. We report a case of a neonatal *B. cereus* bacteraemia complicated by a brain abscess from a neonatal intensive care unit. The neonate presented with bradycardia and desaturations with increased oxygen requirements. Initial blood culture detected *B. cereus* but was considered a contaminant. Repeated culturing of the Gram-positive rod was subsequently considered to be significant. Initial ultrasound head scans revealed echogenicity in the right posterior deep white matter. A large central cavity (5 mm diameter) could eventually be observed. The brain abscess resolved after surgical drainage and an extensive 6 weeks of antimicrobial therapy. This case study describes a rare event that illustrates the importance of rapid identification and susceptibility testing of invasive *B. cereus* isolates from immunocompromised patients.

## Introduction

*Bacillus cereus* is Gram-positive, facultatively anaerobic, toxin-producing endospore-forming bacteria. In human medicine, *B. cereus* is considered an opportunistic pathogen typically as the cause of acute gastroenteritis [1]. In the hospital environment, *B. cereus* spores can be found contaminating a range of hospital surfaces including linen, ventilator equipment and catheters. In the clinical microbiology laboratory, *Bacillus* spp. are often regarded as contaminants when found in blood cultures.

The ubiquitous distribution of *B. cereus* in the hospital environment poses the risk of serious infection to immunocompromised patients [2]. Indeed, systemic *B. cereus*, including bacteraemia with the potential for central nervous system involvement has been described as a risk to patients with neutropenic haematological malignancies [3]. The transmission route in many cases is suspected to be via the presence of contaminated indwelling foreign bodies such as catheters [1]. Neonates and immunocompromised children are another major risk group for *B. cereus* systemic disease, characterized by fulminant infections that lead to high mortality rates. Although rare, the most serious outcome of a *B. cereus* systemic infection is the occurrence of neonatal meningitis and brain abscesses [4]. In these cases, aggressive usage of antibiotics with efficacy for *B. cereus* were associated with a positive outcome [4]. Surgical drainage was also shown to be effective.

From a diagnostic perspective, evaluating the presence of the detection of *Bacillus* spp. is challenging given the presence of this micro-organism in the patient’s environment with the potential risk of contaminating samples for microbiological investigation. In the absence of other risk factors, the detection of *Bacillus* spp. thus requires further microbiological investigation with a high level of vigilance in determining the clinical significance of *B. cereus*, including thorough antimicrobial susceptibility testing. The turn-around time of this microbiological work-up thus becomes a major issue when considering the urgent nature of managing suspected cases of bacterial meningitis.

In the current case study, we detail the microbiological investigation and management of a case of *B. cereus* bacteraemia complicated by a brain abscess from a neonate admitted to the neonatal intensive care unit (NICU) in our hospital in Western Sydney, Australia.

## Case presentation

A pre-term neonate delivered at 30 weeks was admitted to the NICU for prematurity. The baby was delivered following a pregnancy complicated by gestational diabetes mellitus, placental abruption, clinically suspected chorioamnionitis and ante-partum haemorrhage. The neonate was supported by continuous positive airway pressure (CPAP) during the first week followed by high-flow nasal cannula for 3 days. On day 11, the neonate became unwell with frequent bradycardia and desaturations with increased oxygen requirements. Empiric flucloxacillin and gentamicin were commenced after initial blood-culture sampling. A few hours later, the neonate was placed on a mechanical ventilator and commenced on vancomycin due to further clinical deterioration.

**Fig. 1.**
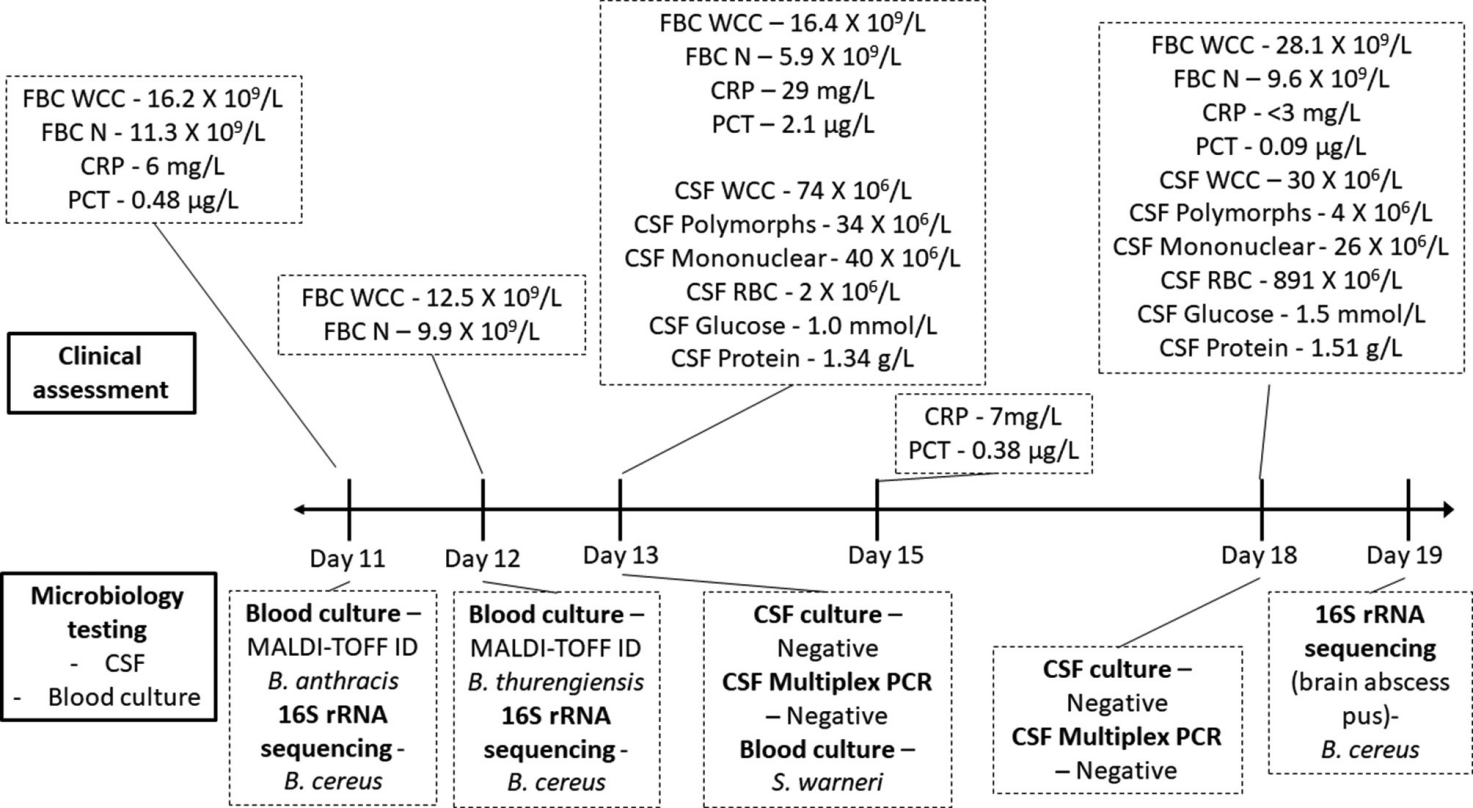
Timeline of clinical assessments and microbiologist testing results for the case.

The timeline for the results of investigations and management of the case is summarized in Fig. 1. A pediatric blood-culture bottle was positive for a large Gram-positive rod within 24 h of initial sampling. MALDI-TOF mass spectrometry analysis (Bruker Daltonics, Germany) identified the organism as *Bacillus anthracis* (score 2.1). This organism was considered as a contaminant, particularly after noting the absence of long lines or indwelling catheters. While it was noted that white cell counts (WCC) were high, C-reactive protein (CRP) and procalcitonin (PCT) results were not particularly suspicious. A repeat blood culture (day 12) was taken prior to switching the empirical antibiotic treatment to vancomycin. The second blood culture taken on day 12 returned a second result for a Gram-positive rod with the isolate later identified as *Bacillus thurengiensis* with a MALDI-TOF mass spectrometry score of 2.1. The repeated isolation of *Bacillus* species was considered as significant. Limited antibiotic susceptibility testing was performed for penicillin and vancomycin E-tests (Biomerieux, Murrarie, Australia) revealed minimum inhibitory concentrations (MICs) of 4 µg ml^−1^ (resistant) and 3 µg ml^−1^ (susceptible), respectively. Based on this testing, vancomycin was continued for bacteraemia due to *Bacillus* spp.

The routine head ultrasound performed on day 11 revealed an area of echogenicity in the right posterior deep white matter, which was not present on the previous head ultrasound. A new finding of echogenicity on the head ultrasound and two positive blood cultures prompted investigations to exclude infective causes for meningitis and/or a brain abscess. Analysis of cerebrospinal fluid (CSF) collected by lumbar puncture on day 13 (Fig. 1) revealed pleocytosis, elevated protein levels (1.34 g l^−1^; normal range 0.15–0.45 g l^−1^) and low glucose levels (1.0 mmol l^−1^; normal range 2.2–3.9 mmol l^−1^). Screening of CSF by real-time multiplex PCR for a range of bacterial and viral pathogens (Biofire FilmArray ME panel, Biomerieux) failed to detect any positivity (*Bacillus* spp. are not included in the panel). The CSF was also negative for *Toxoplasma gondii* by PCR, while a rectal swab tested negative for enteroviruses by PCR.

On day 14, a third blood culture and subsequent MALDI-TOF analysis detected a *mec*-positive *Staphylococcus warnerri* (MALDI-TOF score 2.0). Considering all three blood cultures yielded a common contaminant-like organism sensitive to vancomycin, at this point, a decision was made to continue intravenous (IV) vancomycin for a total of 10 days. The next day (day 15), the infant was noted to have increased respiratory secretions. Culturing of endotracheal aspirate yielded light growth for a beta-lactamase negative *Haemophilus influenzae*. A 5 day course of IV ampicillin was also added.

**Fig. 2.**
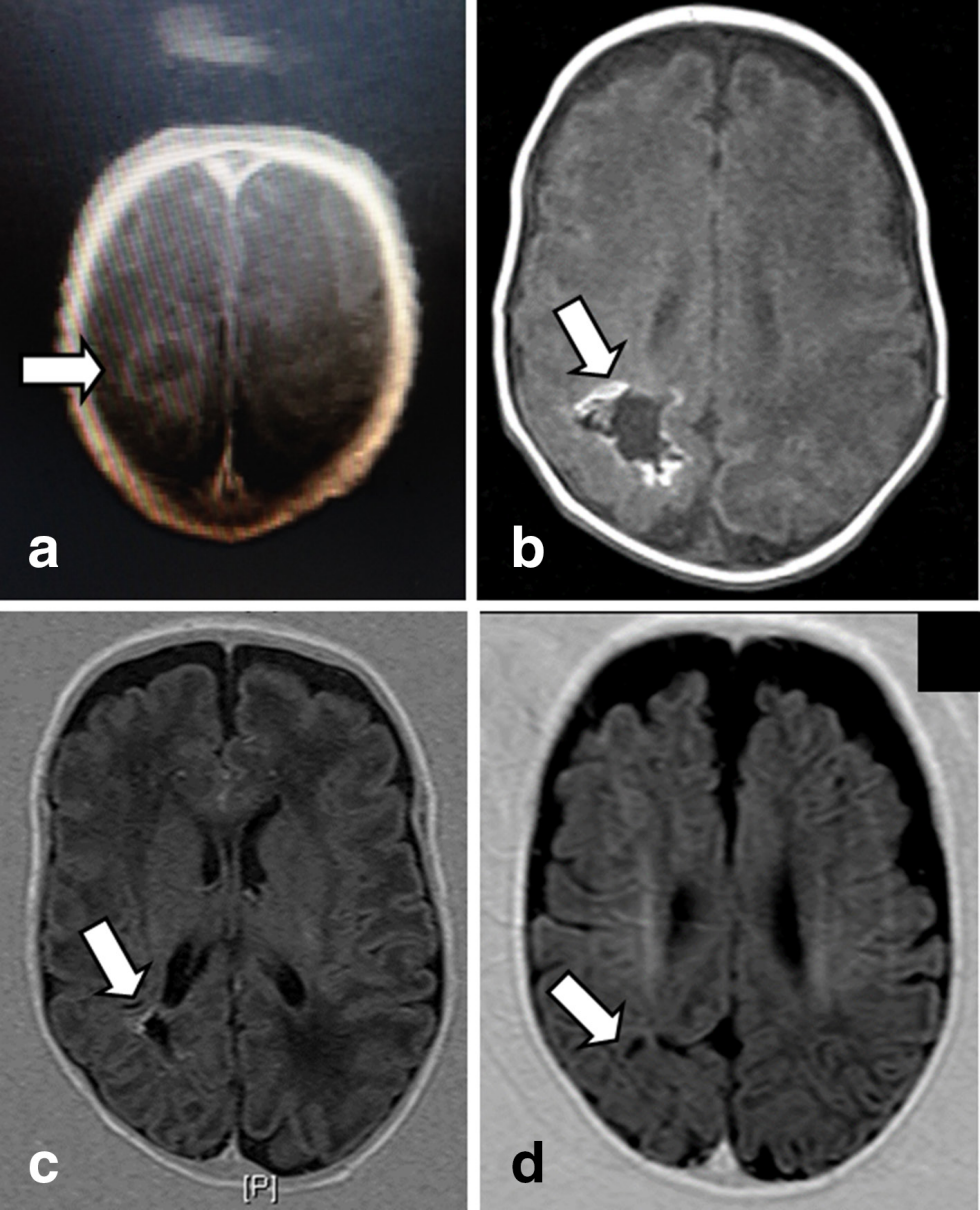
MRI of the infant’s brain abscess. Area of suspected brain abscess indicated by white arrows; (a) day 18 following admission to NICU; (b) 3 weeks after therapy; (c) 6 weeks after therapy; (d) 5 months after therapy.

Lumbar puncture was repeated on day 18 in the NICU. The CSF biochemistry and cytology measurements continued to be within the abnormal range. By this time, the area of echogenicity visualized in the initial head ultrasound scan had progressed to develop a large central cavity with an approximate diameter of 5 mm, suggestive of an abscess, which was confirmed with magnetic resonance imaging (MRI) brain imaging as illustrated in Fig. 2. The infant was subsequently transferred to a pediatric neuro-surgical unit, where the abscess was drained immediately. The abscess fluid was cultured but did not grow any bacteria, although 16S rRNA eubacterial PCR and sequencing detected *B. cereus* rRNA. During this period, extended MICs performed on the original bacteraemic blood-culture isolates revealed the following sensitivity profiles assessed according to the Clinical Laboratory Standards Institute guidelines for fastidious organisms [5]: penicillin (4 µg ml^−1^; resistant), cefotaxime (32 µg ml^−1^; intermediate resistance); vancomycin (3 µg ml^−1^; susceptible), meropenem (0.064 µg ml^−1^; susceptible) and linezolid (1 µg ml^−1^; no interpretation criteria). As meropenem had the lowest MIC, this antibiotic was administered post-surgery for a period of 6 weeks. Full blood counts and radiology (Fig. 2) were used to monitor the patient till the brain abscess was completely resolved. At the 14 month check-up, the child’s development appeared normal.

## Discussion

This case study presents a rare case of a neonatal brain abscess caused by *B. cereus*. As will be discussed, clinical improvement was observed in this case as a likely response to the (i) use of imaging to complement microbiological testing; (ii) early neurosurgical intervention and (iii) timely antimicrobial intervention. Infections in other patients can lead to rapid death or severe and long-lasting neurological sequelae [6].

The initial identification of *B. cereus* and follow-up microbiological testing described in this case study highlight the diagnostic challenges in preparing an appropriate and rapid response to this result, particularly in cases where no foreign bodies were present. As noted previously [4], the use of appropriate imaging technologies was critical to understanding the extent of the infection/disease, supporting the diagnosis of *B. cereus* but also indicating the need for rapid surgical intervention, particularly when the results of microbiological testing of CSF such as that described in our study revealed inconsistent results. In terms of the microbiological investigation, while MALDI-TOF analysis of the *B. cereus* isolates was helpful in providing a rapid identification of the *Bacillus* spp. involved, identification was not perfect with at least one isolate identified as *B. anthracis* rather than *B. cereus*. It is anticipated that this incorrect profile was the result of a database upgrade related to capture of organisms related to ‘Biosecurity’ during this period, to support ‘improved’ identification of *B. anthracis* over other *Bacillus* spp. The failure to consistently detect *B. cereus* across the range of clinical specimens collected from the neonate, otherwise highlights the importance of having a range of traditional clinical microbiology approaches such as culturing, complemented by molecular methods that have a broad scope of detection, in successfully diagnosing an infection such as that described in this study.

The empirical therapy of vancomycin and meropenem is consistent with previous cases reported of *B. cereus* brain abscesses in infants 4, 7] with the isolates in our study sharing similar antibiotic resistance profiles to those described by Horii *et al*. [7]. Surgical drainage was also likely to be significant in the treatment of this individual with a combination of drainage and antimicrobial therapy recommended for the treatment of individuals with brain abscesses [4].

In terms of improving the diagnosis of *B. cereus*, this case report illustrates that the phenotypic identification of *B. cereus* strains is laborious and complicated by the ubiquitous nature of this organism in health-care settings, even when they are detected in vulnerable patients. The development of specific and discriminatory molecular tests would be a solution however this is not necessarily a simple exercise with major questions about whether highly pathogenic strains of *B. cereus* exist (compared to those regarded as contaminants) or whether infection in immunocompromised at-risk populations is simply the failure of the host to mount a sufficient immune response. An environmental reservoir of organism is suspected in our case given the frequent identification of *B. cereus* as a part of environmental testing performed as a part of a concurrent outbreak of *Serratia* in the NICU (data not shown). In the absence of tests to discriminate pathogenic types, molecular tools such as those recently described [2] would be useful to profile the local molecular epidemiology of *B. cereus* strains in a given health-care setting. Such information could then be used to guide clinical judgements in an effort to improve care of patients infected by this challenging bacterial pathogen.

### Consent to publish

Written parental consent was provided to publish the details of this case.
